# Extracting body function information using rule-based methods: Highlighting structure and formatting challenges in clinical text

**DOI:** 10.3389/fdgth.2022.914171

**Published:** 2022-09-06

**Authors:** Guy Divita, Kathleen Coale, Jonathan Camacho Maldonado, Rafael Jiménez Silva, Elizabeth Rasch

**Affiliations:** Rehabilitation Medicine Department, National Institutes of Health Clinical Center, Bethesda, MD, United States

**Keywords:** natural language processing, information extraction, body function, ICF, document decomposition

## Abstract

This paper describes the identification of body function (BF) mentions within the clinical text within a large, national, heterogeneous corpus to highlight structural challenges presented by the clinical text. BF in clinical documents provides information on dysfunction or impairments in the function or structure of organ systems or organs. BF mentions are embedded in highly formatted structures where the formats include implied scoping boundaries that confound existing natural language processing segmentation and document decomposition techniques. This paper describes follow-up work to adapt a rule-based system created using National Institutes of Health records to a larger, more challenging corpus of Social Security Administration data. Results of these systems provide a baseline for future work to improve document decomposition techniques.

## Introduction

Body functions (BFs) are the physiological or psychological functions of body systems (1). BFs are mentioned in the clinical text when there is a concern for, or documentation of, impairments related to body structure or function. BF information is commonly collected during physical exams to provide insight into potential dysfunction within underlying body systems or structures.

Our motivation came from a request from the Social Security Administration (SSA) to retrieve BF mentions within their documents to support existing efforts to enhance their disability claims adjudication process. We are motivated to work on this task as a mechanism to improve the algorithms that support BF extraction, namely, sectionizing, sentence chunking, and context scoping annotators using BF mentions as the use case (2). BF mentions are often embedded in complex formatted text in the form of lists, slot values, and oddly punctuated sentences in clinical notes. This paper reports on the systems developed to capture this information before improving the document decomposition tasks.

Our conceptual framework for BF comes from the International Classification of Functioning, Disability and Health (ICF) (3). While there are many specific kinds of BFs, we set out to find mentions of *strength*, *range of motion (ROM)*, and *reflexes* because of their relevance to the current disability adjudication business process. Within these mentions, we label the BF **type** (strength, ROM, reflex), the body **location**, and any associated **qualifiers**. The ICF does not have atomic level concepts for the BF types we are interested in, so we associated the types with corresponding Systematized Nomenclature of Medicine (SNOMED) identifiers, *44432004* for strength, *364564000* for ROM, and *87572000* for reflex.

## Prior work

We priorly reported on work to extract BF using Biomedical Translational Research Informatics (BTRIS) records from NIH Clinical Center Notes (2). Otherwise, there is little prior work specifically extracting BF from clinical notes. Some work has been done extracting other ICF-defined areas using traditional rule-based techniques and deep learning methods. Kukafka, Bales, Burkhardt, and Friedman report on modifying the MedLEE (Medical Language Extraction and Encoding) System to automatically identify five ICF codes from Rehab Discharge summaries (4). Newman-Griffis and Fosler-Lussier describe linking physical activity reports to ICF codes using more recent language models and embeddings (5). Table 1 outlines the prior work done using ICF constructs.

**Table 1:** Prior work extracting ICF constructs.

Authors	Entities extracted	Data	System components
Divita et al.	ICF codes: • Strength (b730) • Range of Motion (b710) • Reflex (b750) Qualifiers o Body location o Function qualifiers	Physical Therapy/Occupational Therapy notes NIH Clinical Center Data (Biomedical Translational Research Informatics (BTRIS)) records	V3NLP Framework/Sophia/UIMA
Kukafka, Bales, Burkhardt and Friedman	ICF codes: • b117 (intellectual functions) • d420 (transferring oneself) • d530 (toileting) • d550 (eating) • d5400 (putting on clothes)	Rehabilitation discharge summaries	MedLEE ((Medical Language Extraction and Encoding)
Newman-Griffis and Fosler-Lussier	ICF codes: • d410 (Changing basic body position) • d415 (Maintaining a body position) • d420 (Transferring oneself) • d430 (Lifting and carrying objects) • d435 (Moving objects with lower extremities) • d440 (Fine hand use) • d450 (Walking) • d455 (Moving around) • d460 (Moving around in different locations) • d470 (Using transportation) • d475 (Driving)	Physical activity reports from NIH Clinical Center Data (Biomedical Translational Research Informatics (BTRIS)) records	Automated-ICF-coding

ICF, International Classification of Functioning, Disability and Health; NIH, National Institutes of Health.

The NLP platform employed for this work was adapted from the V3NLP Framework (6) and Sophia (7), which were used for symptom extraction and finding mentions of sexual trauma in veteran clinical notes. The framework employed is built upon UIMA (Unstructured Information Management Architecture) (8), so it resembles cTAKES (clinical Text Analysis and Knowledge Extraction System) (9) closely but has a pedigree from the UMLS (Unified Medical Language System) concept extraction in biomedical literature (MetaMap) (10).

## Corpus and manual annotations

### Corpus description

We worked with claimant documentation received by the SSA. Those applying for SSA disability benefits are, within the SSA, referred to as claimants. Claimants' records include evidence of impairments and evidence that they have not been able to work for at least a year because of their impairments. These were, for the most part, medical evidence records (MERs), consultative exams (CEs), and a small amount of other clinical document types. The pool of documentation from which we sampled came from a data pull in 2019 of 16,000 adult disability claims with allegations of musculoskeletal, neurological, or mental impairments, a decision issued during 2013–2018 and spanning five geographically diverse regions of the United States. Records had a PDF structure encompassing a case or part of a case, with the PDF including documents related to that case; 65,514 PDF files in all. Many documents in this collection included notes from multiple clinical encounters over time with a particular healthcare provider (2).

Of the PDFs we received, 50% were categorized as CEs, 40% were marked as MERs, and 10% were marked with more specific clinical document types, but not one category of document type accounted for more than 1% of the total. In fact, all PDFs included at least a cover sheet page and most of the content within the PDF were documents of various but uncategorized types, including progress notes, discharge summaries, Subjective, Objective, Assessment and Plan (SOAP) notes, labs, electrocardiograms (EKGs), radiology reports, and the like. SSA provided only broad categorizations at the PDF level of aggregation, with most of those being either CEs or MERs. While typical NLP tasks preselect document types, we have no current mechanism to decompose the PDFs into the more traditional clinical NLP-categorized document types and no current mechanism to usefully categorize the pages we process.

Sixteen thousand PDFs had been converted to text with Optical Character Recognition (OCR) software by SSA as part of their workflow, resulting in 2.6 million text pages. The quality of SSA's OCR output was not uniform. The resulting text definitely depended on the quality of the image and the formatting. Poor, light, grainy images, images from crumpled paper, images from paper with handwriting scribbled on it, and images from pages with stamps were all causes for poorly OCR’d text. Highly stylized, multicolumn formatted page outlays also fared poorly in that the text recognized was not in useful ordering. Per SSA guidance, cases involving alcohol and drug use were scrubbed, leaving a corpus of 2.5 million pages. From this point on, we treated the pages randomly, with no connection back to a claimant. (Thus, our corpus consisted of pages as the unit of analysis, rather than the claimant or the claim.)

### Sample selection

From the page corpus, we first selected pages that were cleanly OCR’d using principal component analysis (PCA) that encompassed enough attributes to usefully cluster and filter out poorly OCR’d pages (11). The PCA analysis included 62 features that have some latent structure information about each line of the page including if the line starts with an upper case, the case of the first tokens, how many white space characters before the first token of the line, and if the first token is indented from the prior line or not. We included features that counted how many punctuation and number characters were in each line. We included features to encapsulate section or topic shifts à la Marti Hearst's Text Tiling technique (12). The first two principal components from each page, when graphed in a Cartesian coordinate grid, clustered pages that shared similar structural features into areas of the graph. For instance, poorly OCR’d pages were clustered to the right of the graph. Pages that were medication lists or vitals were clustered in the lower right part of the graph. Pages that included long paragraphs as would be seen in consult notes were clustered in the upper right of the graph. Pages that had a mix of structures, well OCR’d, as would be seen in History and Physicals, were clustered around the origin of the graph.

Next, we selected pages that had some indication of BF within the page. We looked for matches using a list of 2,885 fully inflected BF term forms. What we defined as a BF term came from lexicons developed from prior work. We pulled terminology from relevant UMLS sources and terms observed in annotated NIH rehabilitation notes described in (2). All 2.5 million pages were ranked by the frequency of a number of BF terms within them. We then created a stratified sample from the ranked set. The goal was to include in our sample a diverse set of document types where some of the documents might include a few mentions and some documents were all about BFs. We did not want to only sample rehabilitation progress notes, a note type that would likely include the most BF information. Rehabilitation progress notes are more uniform in language and structure in how BF mentions were documented than other kinds of documents. While we made sure some rehabilitation progress notes were represented in our draw to ensure we could adequately process them, we did not want to skew our sample to this note type. Our draw included 500 pages such that every page had at least two BF terms within the text.

### Corpus sample characterization

Pages in this sample had on average 2,212 characters and 388 words per page; see training/testing split outs in Table 2.

**Table 2:** Distribution of manual annotations in SSA records.

Annotation type	Training	(Mean per file)	% of total annotations	Std	Testing	(Mean per file)	% of total annotations	Std
Files	357				90			
Annotations	6,752	17.7		20.5	1,907	19.7		22.54
BF mention	1,541	4.3	22.9	4.6	464	5.1	24.3	5.8
Strength	641	1.8	9.5	2.8	167	1.8	8.8	3.1
ROM	872	2.4	12.9	4.2	261	2.9	13.7	4.7
Reflex	253	0.7	3.8	1.5	59	0.6	3.1	1.0
Body location	1,250	3.5	18.5	4.6	309	3.4	16.2	4.7
Qualifiers	1,745	4.9	25.8	5.7	514	5.7	27.0	6.6
BF context	387	1.1	5.3	1.5	133	1.5	7.0	1.8
Possible BF	63	0.2	0.9	0.6	27	0.3	1.4	0.8
Chars per file		2,228.5		655.1		2,196.8		566.0
Lines per file		83.0		31.1		82.9		28.5
Tokens per file		389.2		116.8		387.0		105.4

BF, Body function; SSA, Social Security Administration.

**Figure 1:**
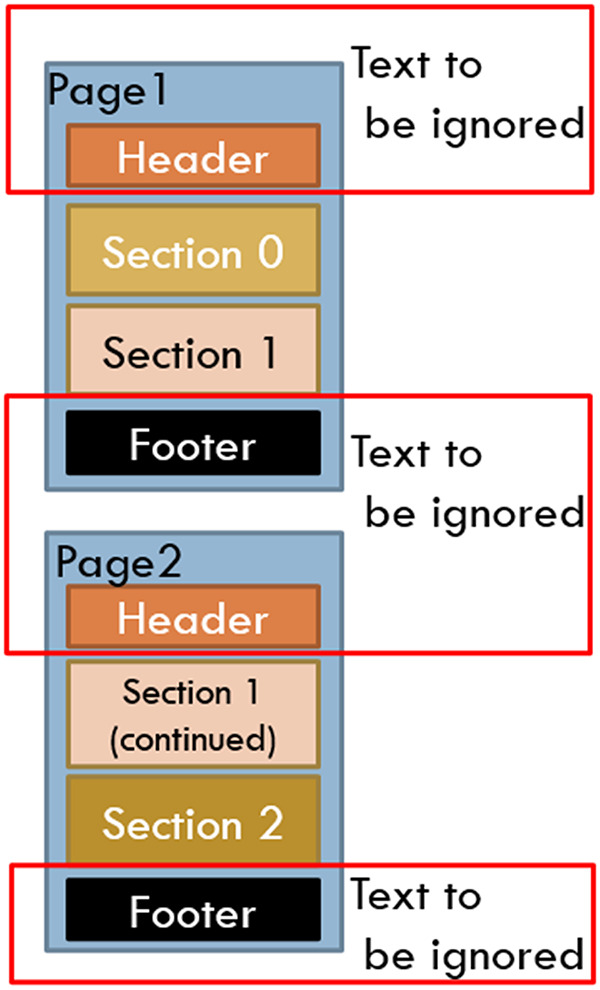
OCR and format challenges: page headers and footers.

**Figure 2:**
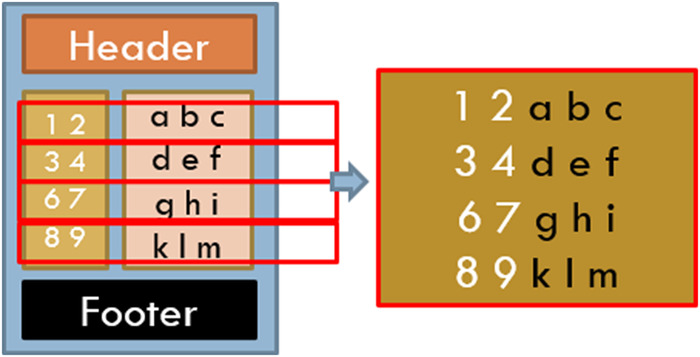
OCR challenges: multicolumn text munged.

However, there was a large variability across the pages chosen in the number of words, lines, and words per page. These pages also had novel characteristics not commonly found with other NLP tasks, in that many of these pages had page headers and footers that needed to be dealt with (see Figure 1) and that pages often included partial sections either from the page before, or to be continued on the page after. Also, complicating matters were idiosyncrasies caused by OCR behaviors. Anything formatted by tabs or multiple spaces, multiple columns, or tables was munged into text in an unreliable, unordered, and often unreadable text (see Figure 2). OCR misspellings were also present and created confusion but were less of an overall issue. We were able to annotate 447 of the 500 records, split into approximately 80/20 sets with 358 training pages and 91 hold-out test pages.

### Guidelines and manual annotations

**Figure 3:**
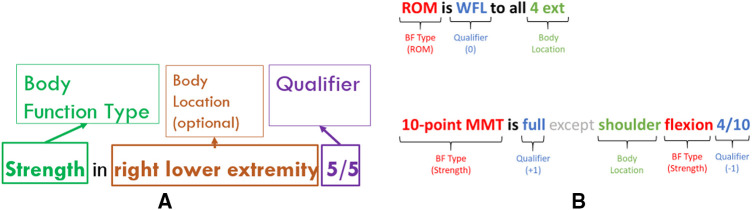
(**A**) Fully formed body function mentions. (**B**) Examples of body function mentions in the clinical text.

Our annotation guidelines (see Supplementary Appendix: Body Function Guidelines) specified that a BF mention is identified when there is a mention of *body function type*, a *qualifier*, and optionally, one or more *body locations* within the scope of a phrase or sentence (see Figures 3A,B). These mentions were only annotated from objective (clinician-observed) information. No information related to treatment planning was considered, as this information does not necessarily speak to an individual's ability to perform certain tasks from an objective measurement perspective.

Laterality and similar modifiers were to be lumped with body location, as BF locations are typically modified with descriptors such as left, right, both, proximal, and distal.

**Figure 4:**
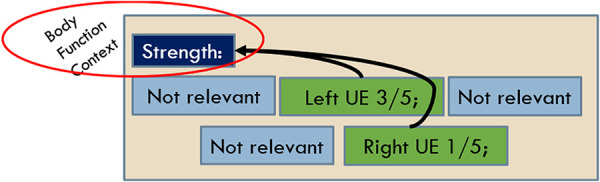
Body function context annotation.

The guidelines were augmented beyond the prior work to accommodate SSA-specific situations including some pages where a part of the page was poorly OCR’d, with some BF terms included in the poorly OCR’d portion. In an effort to lessen the cognitive load on the annotators to put together partial BF mentions from munged text, we included a *Possible Body Function* label to mask out mentions found in that area on the grounds that they are not readable, interpretable, or trustworthy at face value. We also added a label to mark useful *Body Function Context* to accommodate the creation of a mention where the parts of that mention were discontiguous, often seen as a section heading like *Strength*, where the specific strength BF observations are embedded in that section along with other non-strength observations such as ROM or coordination (see Figure 4).

This corpus also included additional kinds of reflex data not specifically observed in the NIH data around plantar reflexes, as they are usually standard practice to be documented as part of the neurological component of a physical exam (PE). Likewise, eye movements were not specifically observed in the NIH data but are now marked in the SSA corpus. Extraocular movement, an objective statement of strength and ROM of the ocular/eye muscles, is related to vision and dizziness in the PE and is often a sign of either a cranial nerve impairment or brain impairment but manifests as a muscle weakness so fits the schema for strength and ultimately has a significant functional impact when impaired.

### Tale of interannotator agreement

The sample was annotated by two experienced medical practitioners. In our effort to ensure that they were consistent between themselves, a pre-established level of interannotator agreement was reached before the corpus was annotated. Three rounds were necessary to solidify the guidelines and give enough experience to the annotators before the inter-rater reliability was sufficiently high to have them independently annotate pages for maximum productivity. We chose a threshold of 0.70 agreement between the two annotators to be achieved before proceeding to annotate the corpus independently. See Table 3 for the inter-rater agreement between the annotators.

**Table 3:** Inter-rater agreement between two annotators.

Round	Macro F1	Body function F1
1	0.52	0.38
2	0.57	0.52
3	0.77	0.71

**Figure 5:**
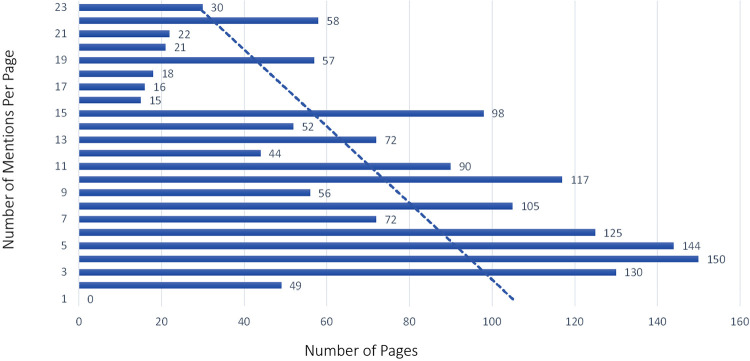
Body function mention frequency training sample by page.

**Figure 6:**
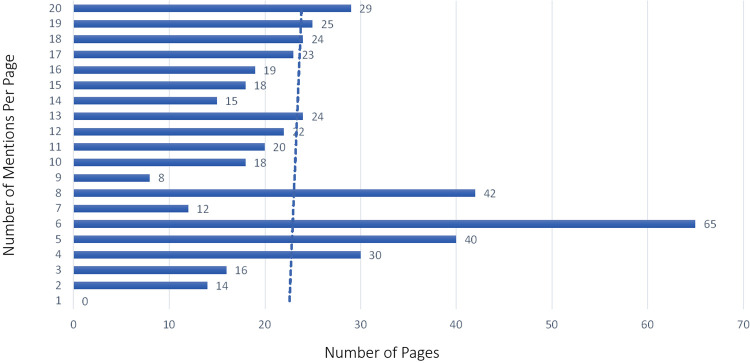
Body function mention frequency testing sample by page.

**Figure 7:**
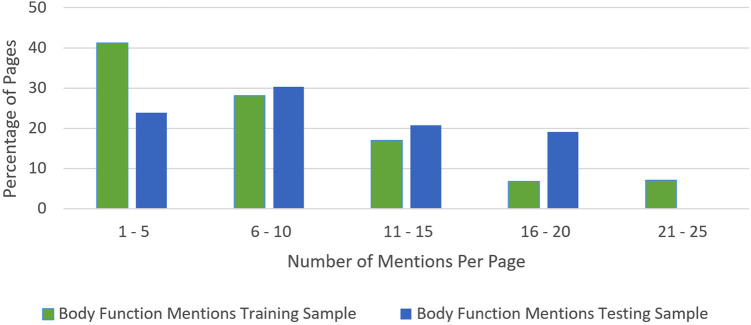
Body function mention frequency percentage.

The majority of pages in the training set had between 3 and 5 BF mentions, with a maximum amount of 23 mentions on some pages. While we tried to get a uniform distribution across pages with a few and many mentions, the distribution was more weighted toward pages with a few mentions in them in the training set by happenstance, as seen with the trend line in Figure 5. The testing set did have more of a uniform distribution across the frequency range (see Figure 6). The percentage of pages that fall in 1–5, 6–10, 11–15, and 16–20 mentions per page is different between the training and testing set in that the training set is skewed to having pages with fewer mentions, whereas the testing set clumps more pages that have between 6 and 10 mentions per page than the other bins (see Figure 7).

## Methods

The BF extraction work relied upon a rule and dictionary-based pipeline approach, built from components from the V3NLP Framework, now rebranded as Framework Legacy, a UIMA-based suite of functionalities. The pipelines in use are stitched together (mechanical annotators) to decompose the clinical text into its constituent parts, including sections, sentences, phrases, tokens, and dictionary looked-up terms. The intelligence of the system used a dictionary lookup annotator, which relied upon lexicons to identify BF mention components. The pedigree of each of those lexicons is described by Divita et al. (2). The pipeline is described here in Supplementary *Appendix: Body Function Pipeline Explained*.

The pipeline as described by Divita et al. (2) was augmented by the addition of a (machine) annotator to assign the polarity of qualifiers, modifications to capture more mentions by using section-level context, and additions to the lexicons.

### Lexicons

Lexicons were developed for Strength, ROM, Reflex, Body Location, and term-based qualifiers applied to BF. Lexicons were developed to mask items that could be confused with BF but were not, including a pain lexicon and a balance and coordination lexicon. The sources to each of these started with a traversal through UMLS terminologies, where top-level relevant terms were identified and algorithmically, hierarchically decedent, and related terms were extracted. These terms were augmented by lexical variants using NLM's Lexical Variant Generation (LVG) (13) to create fruitful variants (14). The entries in each of the lexicons are tagged or categorized with either Strength, ROM, Body Location, or the like. Metadata including the UMLS identifiers and UMLS semantic types were retained for pedigree's sake. The information contained in these lexicons constitutes the information used by Named-entity Recognition (NER) when a mention is found.

#### Additions to the lexicons

Once we had annotations for the SSA corpus, additional terms were added based on a review of manual annotations from the SSA training corpus that were not covered by the existing dictionaries. Additional terms were added to the nonbody function/confounding term lexicon to filter out false positives. There were 101 additions to the Body Location Dictionary, now with a total of 53,430 indexed entries. No additions were necessary for body strength, body qualifiers, or terms that encompassed an entire mention in one term. The concept for plantar was added to the body reflex lexicon to account for terms seen in SSA data that were not seen in the BTRIS data. There were an additional 101 terms added to the nonbody function or confounding terms to mask out mentions that turned out to be eye vision qualifiers, i.e., 20/20, mobility-related terms, medication dosages, pulse and other vitals, additional pain-related scores, muscle tone, stiffness, alignment, and hearing-related mentions. There were an additional 57 terms added to the ROM Dictionary, now with 798 entries. Two new lexicons were added to replace what had been a set of rules to define allowed sections to look for BF mentions (134 entries) and 73 filtered out section names to ignore mentions that fall within these sections.

### Preprocessing annotators

The original work required some preprocessing to handle overly redacted text and text that had no newlines. The SSA corpus was structured differently in that there are no redactions *per se*, so that part of the pipeline, while not turned off, had no effect on the text. While it has been observed that some of the 2.5 million pages within the SSA corpus also have no newlines, those sampled for the manual annotation training and testing serendipitously had newlines. While the part of the code employed to handle text with no newlines was not turned off, it had no effect on this work.

Some files included two or three pages rather than one page. Some preprocessing was done during the sample selection process to augment the pages where the mention was at the top or bottom of the page to include the page before or after.

### Qualifier polarity assignment

The pipeline as described in Divita et al. (2) was augmented by the addition of a (machine) annotator to assign the polarity of qualifiers. The assignment of a *+1* was given to a qualifier that indicated an at-or-above-level functioning, a *−1* assigned to qualifiers that indicated a below-level functioning, and a *0* for ambiguous and qualifiers where no assignment could be made. These assignments were done by rule to qualifiers in one of two ways. The qualifier polarity annotator cycled through qualifier mentions, and for those mentions that came from term lookup, utilized a qualifier polarity attribute if it was present in the dictionary. For example, dictionary entries for terms like decreased and impaired included a *−1* attribute to be carried along and used. Those qualifier mentions that were identified *via* a dictionary lookup, where there is no polarity attribute, received a value of *0*.

The qualifier attributes in the dictionary were assigned first by observing the manual assignments from the training annotations for both BTRIS and SSA corpora. A manual review of the terms in the qualifier lexicon was also undertaken to assign attributes. Of the 2,413 BF qualifier terms in the dictionary, 483 were assigned −1 and 89 were assigned +1.

For those BF qualifiers that are numeric, simple rules were created within the annotator to assign the polarity.

If the BF was found to be a *Strength* mention, and the numeric mention was in the form of a fraction, and if the fraction equated to 1, i.e., 5/5, 10/10, the qualifier polarity was assigned +1. Otherwise, the qualifier polarity was assigned *a* −1.

A completely unprincipled, empirically based set of rules were formulated to assign polarity assignment for a ROM. Compromises were made in lieu of a complex set of rules where there would have to be a rule made for each location detected for a ROM measurement. It was observed in the training data that only seven ROM cases involved an at or normal ROM (+1) assignment where the qualifier was a degree. In all of those cases, either 45 or 50 degrees were mentioned, and in 4 of the negative cases, 45 or 50 degrees were mentioned. Thus, the rule assigned was only assigned +1 to cases where the degrees were either 45 or 50 degrees, knowing that this rule was flawed and would only miss a few cases.

Reflexes are often measured on a scale from 0 to 4+. In our work, only a score of 2+ (a brisk response; normal) was defined as a *polarity value* equal to +1. Otherwise, *a −*1 was assigned as the *polarity value*. If the BF was otherwise found to be a *Reflex* mention, and clonus was mentioned, the numeric value was assigned a *polarity value* of −1.

### BF context

The BF context mentions were utilized in a limited way by the following: If a qualifier was found, but it did not tie back to enough evidence to make it part of a BF mention, the scope to find such evidence expanded to the left all the way back to section headers. If, within that scope, a section header was found that provided the missing evidence (usually a BF type or BF location), a mention was created from the contiguous evidence around the qualifier (see Figure 4).

### Possible BF

We did not take advantage of the possible BF annotations that marked areas where there would clearly be false positives. The original intent was to mask out these areas from evaluation; however, in retrospect, that would have been cheating and was not done. As a consequence, many false positives were reported from areas that were munged.

## Results

### Rule-based system: token-based matching criteria

See Table 4 for the token-based BF evaluation on the test sample.

**Table 4:** Token-based body function evaluation test sample (results from prior work are in parentheses).

Label	F-1 Score	Recall	Precision	Accuracy
BF mention	0.5871 (0.61)	0.7795 (0.94)	0.4709 (0.45)	0.4489
Qualifiers	0.6018 (0.56)	0.7326 (0.85)	0.5107 (0.42)	0.5494
Type	0.5882 (0.63)	0.6658 (0.88)	0.5268 (0.49)	0.5381
Body location	0.4249 (0.46)	0.4941 (0.82)	0.3727 (0.32)	0.4113

BF, Body function.

### Rule-based system: entity-based matching criteria

See Table 5 for the results for the entity-based BF evaluation on the test sample. See Table 6 for the entity-based BF evaluation on the training set.

**Table 5:** Entity-based body function evaluation (test sample).

Label	F-1 Score	Recall	Precision
BF mention	0.6682	0.8857	0.5364
Qualifiers	0.6327	0.7976	0.5242
Type	0.6257	0.7811	0.5218
Body location	0.4473	0.7281	0.3228

BF, Body function.

**Table 6:** Entity-based body function evaluation (training).

Label	F-1 Score	Recall	Precision
BF mention	0.6616	0.8920	0.5258
Qualifiers	0.6300	0.7983	0.5204
Type	0.5662	0.7313	0.4620
Body location	0.4797	0.7634	0.3497

BF, Body function.

The entity-based qualifier evaluation confusion matrix from the testing sample was noted to be True Positive (*TP*): 410, False Positive (FP): 372, and False Negative (*FN*): 104. Note that our entity-based evaluation did not define what the true negatives (TNs) were, so the table is devoid of *TNs*.

### Rule-based system: polarity evaluation

The testing efficacy of the polarity was reported to be distinctly different than the F1 score. The values were predicated on the machine already finding a mention, to begin with. As such, there were no false positives. What is being reported is a percentage based on the following formula: (*TP*/(*TP* + *FN*)) × 100. This can be thought of as a surrogate for accuracy.

The −1 polarity qualifier (below-level functioning) was correctly identified in 82% of the cases where the machine identified a polarity mention. The +1 polarity qualifier (at or above functioning) was correctly identified in 67% of the cases where the machine identified a polarity mention. The machine only correctly identified the 0 polarity qualifier (ambiguous) values in 43% of the cases where the machine identified a polarity mention.

### Failure analysis

#### Qualifier polarity failures

In total, 13% of the failures can be attributed to the completely unprincipled, empirically based set of rules that cover the ROM polarity assignment. In total, 5% can be attributed to terms that were in the lexicon but did not have a qualifier category assigned to them. Another 5% can be attributed to negation evidence around the qualifier that was not considered or where the scope of the negation was wrong. There were a few cases where the qualifier category in the lexicon was just wrong. An example of this was the qualifier “down going,” categorized as −1 when it should have been 1. Another 7% can be accounted for by the machine-labeled qualifier correctly, and the gold standard mislabeled the qualifier.

#### Body location false negatives

Body location was the least successful extraction of the endeavor. A review of the FN location mentions indicates the following insights. While there were mentions missed because of missing entries in the lexicon, this kind of error accounted for 32% of the failures. Those missing entries included consumer-level anatomy terms like *great toe* and *eyelids* and a few misspellings like *musculoskeletal*. A class of acronym spellings was absent, and even if they were present in the lexicon, they would/do cause some consternation because at least one overlaps with a preposition. While (full range of motion) *F-ROM* was in the lexicon, *FROM* was not a spelling variant, and even from the documented context, a Part-Of-Speech (POS) tagger would have tagged the fragment as a preposition rather than the intended noun phrase.

#### More failures explained

There is a rule to filter out extracted entities within spans that are historical, not related to the patient, hypothetical, or conditional. A noted number of missed locations turned out to be filtered out because the body location was within the scope of historical evidence. In theory, if *a history of a decreased ROM* was documented, it would not be marked because it is not a current objective mention. A historical evidence annotator was used to find and mark historical evidence. It is overzealous in doing so, marking absolute event dates within the scope as historical evidence and filtering out many of the BF mentions, including body locations. A more constrained notion of history is called. This was caught in 4 of the first 15 false negatives found. A broader census of the failure was not possible due to the length of time focused on each instance. Its scope will be better known once the issue is fixed and a re-evaluation is done.

There was a class of failures that could not be easily ameliorated. Among them, OCR errors that delete spaces between words caused section names to be missed, terms to be missed, and the like. For example, section context was missed due to such an error, where the snippet included *toes*. *Reflexes Exam*.

There was a class of failures where the location part of terms was in the lexicon as part of a specific strength or ROM mention such as in *plantar flexion* (C0231784), but these were not also labeled with location semantic categories. There were gaps in the effort to have all semantic categories marked in each lexicon that each term covered. As a result, some terms like *plantar flexion* were not also marked as *body location*. A more thorough review is needed to find and label body location in the strength, ROM, and reflex lexicons. At least 4% of the cases of failure were attributed to this kind of error.

**Figure 8:**
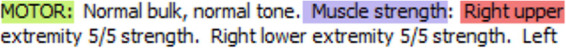
Scoping error example.

There were a host of errors caused by poor scoping. At least 2% of the cases involved where a section or slot value name started in the middle of a line. The section name annotator is rigid in labeling only section names that start at the beginning of the line. Because of this, there were scope failures. For example, in Figure 8, *MOTOR* was marked as a section name, and Muscle strength was marked erroneously as a slot value. In general, slot values do not cross line boundaries and do not end in a period. The scope of the construction ended at the end of the line rather than in lines to follow. Consequently, the *right upper extremity* was not recognized (see Figure 8).

**Figure 9:**
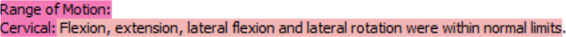
Ambiguous section name, slot value structures.

There were related scoping issues caused by interpreting a heading as a section name rather than a slot heading that caused a body location to be missed. Section names provide context but only alert the reader that useful information is coming. Slot headings, on the other hand, are questions asked, where the answer is in the text to the right of the colon delimiter. The contents of a section name do not directly participate in a mention other than to provide some form of context. Slot headings participate in BF mentions. In Figure 9, *Cervical* was labeled as a section name. However, it really was a slot value, providing the location portion of the ROM mention that followed.

#### False positives

Body location was the worst performing annotator in terms of false positives, so it warranted review before the others. Fifteen percent of the false positives came from laterality terms, as laterality also appears in body structures and BFs that were not strength, ROM, and reflex. The top FP words included *bilaterally*, *neurological*, *right*, and *left*.

A number of those were within straight leg raise (SLR) mentions that, upon consultation, are not considered BF but had been erroneously added to the ROM lexicon as part of the initial effort to pull terms from the UMLS related to ROM. A number of FP mentions were exercises to do within sections that were not labeled as plan sections. This has inspired the addition of *reps*, *training*, and *exercise* to be added as future confounding terms. Otherwise, there were no large categories to pinpoint. A number of body location mentions and BF mentions came from sections that should be added to the filter-out list. Most of these sections were spelling variants or synonyms of existing section names. Among them are *Rx*, *Chief Complaints*, Subjective and Objective (*SO*), *Claimant Alleges*, *Plan of Care*, and *History*. From the category of “lacking face validity,” it was noted that “normal strength” showed up as body location false positives. When tracked down, the lexical entry came from a synonym of *muscle strength normal* (C1836901), which could infer a body location. Also noted is that a number of FP body location and BF mentions came from mentions that were within conditional phrases. Tracking this down, filtering out body locations within conditional phrases had been turned off/commented out during the tuning of the application to the NIH BTRIS data. Performance was worse with that dataset when conditional phrases were filtered out.

We do not currently yet have the tooling to identify which false positives fell within the bounds of those munged possible BF areas, but a vast number of otherwise unexplained false positives are coming from these munged areas upon manual review.

As noted in the prior paper, a significant number of false positives were, upon manual review, true positives. At this scale, and amount of OCR-induced noise, it is expected that some mentions get missed. This has also been true of the SSA dataset. A number of true positives have been identified from the FP list, and consequently, the gold standard set is being updated.

## Discussion

Among the limitations of our approach, we recognize that we were compromising by choosing to adopt a completely unprincipled, empirically based set of rules, essentially what amounted to a manual logistic regression to maximize the polarity positive cases and minimize the negative cases using as few attributes as possible. We chose this approach due to the burden of building a series of rules for specific body parts for the few number of cases we had such that the investment was not worth the return. This may have had the effect of reducing our efficacy with this dataset but also possibly prevented overfitting.

In comparison with the prior work, the system, with the aforementioned additional tuning, did well when turned to a much more complex, noisy corpus. Precision overall went up at the cost of recall across the board.

We did not build the application with speed or performance as a requirement. Our application processed 447 pages in 1 min and 46 s on an admittedly fast NVIDIA DGX1. However, the application is built from UIMA components. This application, built upon the UIMA framework, can be configured to parallel process and scale-up and scale-out the pipeline to address performance and throughput criteria if needed.

The tool is built from UIMA components, and although run using one admittedly fast NVIDIA DGX1, running at 1 min 46 s on average per run on all 447 records, the tool can be configured to be multithreaded to run concurrently to scale up process faster if needed.

While annotations were used to (manually) learn from, there are no opaque models which might contain sensitive information contained within the source code. This tool should be devoid of any privacy issues when time to distribute the application comes.

Test results on the training set are not normally acknowledged; however, as part of the story about this being follow-on work from a system initially trained and tested on NIH data, it is worth noting that there was little performance degradation between the training (see Table 6) and testing (see Table 5) runs. This is thought to be a telling success in the detail we went through to ensure that the training and testing samples were as similar as possible.

We chose to take a rule-based approach based on the domain that embeds meaning in highly formatted, telegraphic language, not at all similar to pretrained language models available to us at the time. Also, we did not have enough manual annotations to adequately learn and evaluate to cover the domain. We could and did take advantage of existing standardized vocabularies that do cover most of the domain, enabling us to cover what might not have been in the training set but seen in the testing set. The pretrained language models would not have done so due to the lack of exposure to either the terminology or formatting styles that enable the learned relationships.

## Future work

Insights from the failure analysis will be folded into the codebase. The source code and executable jar files will be made available from https://github.com/CC-RMD-EpiBio/bodyFunction.

This tool will be retooled using statistical machine learning models around document decomposition functionality from annotations. That work aims to demonstrate the utility of better segmentation functionality on NLP tasks such as extracting BFs.

If there is a need, this work can be easily expanded to include additional BF types such as balance, coordination, hearing, and muscle tone.

## Conclusion

This work described work to extract BF mentions containing strength, ROM, and reflexes from a large heterogeneous and noisy corpus of clinical pages that had been OCR’d. We noted efforts to create a uniform, representative sample stratifying by BF word frequency within each page of a larger corpus of 2.5 million pages. We described the manual annotation task that created a training corpus and testing sample that was as representative of the overall corpus as possible. We noted functionality added to the original tool as we deployed the tool on this more challenging corpus. The results are encouraging, with improved precision at only a small cost of lower F1 scores across the extracted entities.

## Data Availability

The data analyzed in this study is subject to the following licenses/restrictions: The data used for this study came from the Social Security Administration. Our organization does not have permission or rights to share it. Our organization has no intellectual property rights or privileges to the data we were given access. In addition, this data contained sensitive information including PHI and PII, which required additional security and access safeguards be put into place prior our use of it.
