# Supplementary material for: Extracting body function information using rule-based methods: Highlighting structure and formatting challenges in clinical text
Source: Front Digit Health. 2022 Sep 6;4:914171. doi: 10.3389/fdgth.2022.914171 (PMC9485548; doi:10.3389/fdgth.2022.914171)
Supplement: Supplementary file 1 [file Data_Sheet_1_v1.pdf]

Appendix: Body Function Pipeline Explained

2021-12
Guy Divita

This body of work relies on an NLP UIMA based pipeline that decomposes text into its constituent parts. This pipeline is substantially similar to what was used in the Sophia pipeline, but is outlined here because some additional components have been added. UIMA pipelines are built from a reader, annotators and writers. While there is not much difference between annotators readers and writers, a reader also reads in file or stream and converts it into a construct called a CAS which holds the original file, stand-off annotations that subsequently get created and any other meta-information about each file. Annotators are piped together where the output of one is the input to another. More correctly, the annotators pass along the CAS's which accumulate the annotations or alter annotations made in prior annotators. The annotators noted in this appendix are in the order they are called.

1. Preprocessing Readers/Annotators

1.1 Redaction Annotator/Reader

The redaction done on the BTRIS data was overly aggressive. Templated redacted forms were found within section names and body function mentions as well as other locations. This reader/annotator was created to label redacted forms, to allow those spans of text to become invisible and are temporarily removed for downstream annotators.
A particularity of the BTRIS data set is that redactions are in Section Names such as *History of [First Name id = XXXXX] Illness:* . Taking out the redaction enabled many sections to be correctly identified.

1.2 Add Newlines Annotator/Reader

One of the datasets we work with has a particular idiosyncrasy: it contains no newlines. While not usually worth noting this level of data quality control and normalization, it is noted here because other datasets that come from a variety of providers from around the world also occasionally include documents that have no newlines. An annotator was created to infer when there were no newlines in a document and inject newlines around section names from a rough lookup of section names and simplistic regular expressions. This aided in subsequent section boundary and section name identification because the existing sectionizing mechanism requires newlines to be present.

2. The Syntactic Pipeline

For the most part, the annotators listed here do obvious tasks that need no further explanation. There are exceptions and white lies of course, which will be noted for the seemingly mundane tasks for Tokenization, Sentence Chunking, and Date and Time identification. As it turns out, within the richly heterogenous data we are processing, those tasks are not as straightforward and error-free as is ultimately needed.

2.1 Line Annotator with Blank Lines

This annotator creates annotations for each line in a document. It does not strip empty lines out. Having line annotations enables an algorithm to walk through lines of text. Multiple blank lines indicate a topic shift. Thus, one needs to keep track of those kinds of lines, rather than filter them out, when looking for paragraph breaks. This annotator does not work well when there are no newlines in text, as is the case for the BTRIS data we are using. Special ameliorations are needed for such data.

2.2. RegEx Shape Annotator

The regular expression shape annotator creates annotations for emails, phone, URLs, zip codes and common redaction artifacts found in clinical text. Shapes are pseudo lexical entities, have meaning, but would not normally be looked up in a dictionary, which would distinguish them from lexical entities that come from a dictionary lookup. This annotator Identifies the easy things you don't want which makes the task of identifying things you do want easier. Identifying these entities makes sure that downstream annotators do not erroneously pick up entities that are these.

2.1 Date and Time Annotator

This annotator identifies dates and times via regular expressions.

2.2 Token Annotator

This annotator chunks the text into space delimited units. It creates word tokens and white space tokens. The tokenizer used here also creates attributes describing if the token has punctuation, is only punctuation, has numbers, is only numbers, starts with upper case, is camel case, and ends with sentence ending punctuation. A technical note: this tokenizer is the V3NLP Framework Tokenizer, a tokenizer tuned for clinical text that has a legacy from MMTx and MetaMap.

Tokenizers play an unspoken, but big role in errors downstream, and no tokenizer does a perfect job with clinical text. This tokenizer informally compared to the python based *scispacy* language model driven tokenizer. Both tokenizers had failures with different difficult to parse texts, with neither exhibiting brilliance, one way or another. As a consequence, this legacy version of the tokenizer continues to be used, in great part because it is much faster and has a much smaller memory footprint than the wrapped *scispacy* tokenizer.

2.3 Date by Lookup Annotator

This annotator identifies parts of temporal expressions by items listed in a date lexicon as being a date. These include the obvious – names of the months and days and holiday names.

2.4 Date and Time by Token Annotator

There are oddball dates that get missed by the regular expression annotator before tokenization. This annotator identifies dates that bounded by each token.

2.5 Checkbox Annotator

This annotator identifies and analyzes mentions like *Smoking: yes [ ] no [x]*. The annotator identifies the heading, each of the options, and which option was marked. It identifies whether the options have a positive or negative polarity to them. If so, it takes the polarity of the marked option and applies that polarity to the heading. In this example, *smoking* gets negated because the no box was marked, noting that *no* has a negative polarity.

[Note: This annotator was turned off for this work partly because the BTRIS data did not have checkbox mentions that were relevant to the task.]

2.6 Slot: Value Annotator

The slot:value annotator identifies and analyzes slot and value entities into a content heading entity and an answer entity.

Example: *Denies Alcohol*: *yes.*

Slot:value entities are telegraphic sentences which lack an explicit verb. They are quick methods of data capture and easy comprehension but do not syntactically parse in the same way sentences in prose do. There are a lot of variations to slot: value formats within clinical text in general, and within the BTRIS dataset. Getting this structure correct is paramount. However, there are many ambiguous examples which flummox the current iteration of this annotator.

2.7 Sentence Chunker

This annotator identifies sentences within the text. Embedded within this task, are also the identification of lists and list elements. Like the slot:value annotator, correctly identifying the bounds of when a sentence begins and ends is paramount. The variation of text found in clinical text have flummoxed all the sentence chunkers tried thus far. None have worked 100% of the time. Many of the downstream errors are attributed to sentence chunking failures.

2.8 Term Annotator

This annotator chunks together tokens into terms based on dictionary lookup. Categorization and syntactic information from the dictionary are tagged onto the terms created. The UMLS SPECIALIST Lexicon, by default, is employed to chunk general English into terms. There are annotator specific lexica also employed, including a date lexicon, a lexicon of section names, a lexicon of assertion terms. Most of the pipelines employ 20 lexica of one kind or another.

2.9 Assertion Evidence Annotator

This is one of two annotators that work in conjunction with each other. This annotator identifies evidence for negation, conditional statements, hypothetical statements, whether the mention is about the patient (subject), whether the mention is historical, and who is saying the mention. The algorithm employed is a re-write of Wendy Chapman's ConTEXT algorithm in java. The Lexica came from her rules, and greatly augmented from work done by three groups at the University of Utah combining each group's rules. Who is saying the mention (attribution) is the newest extension to this algorithm and was done for this project. The annotation guidelines stipulated to ignore patient authored statements, thus, the need to identify who is saying what. While it is not completely straight forward to identify patient reported mentions, there are clues or evidence, including trigger statements such as "patient reports", and patient notes". Also, any mentions that come from the subjective portion of SOAP notes are a-priori ruled as patient reported. The rules used for this work were adopted from work done to determine the difference between a sign vs a symptom and work done to determine if the statement is about the patient vs someone else.
Spoiler alert: the second annotator, the assertion annotator, is much further downstream in the pipeline.

2.10 Unit-of-Measure Annotator

This annotator identifies things that are measured, are like terms, but not something to be looked up. These include numeric test results, pulse rates, ejection fractions, or degrees of range of motion. This annotator employs, for the most part, a combination of dictionary lookup for the units part, and regular expression for the numeric parts. The dictionary used for this is a snapshot of NLM'sUCUM resource. Not perfect, but useful.

2.11 Term Shapes Annotator

This annotator identifies spelled out numbers and units of measure ranges.

2.12 Punctuation Terms Annotator

This is a corrective annotator: it creates terms that are only punctuation like +++. The current lexical lookup ignores runs of only punctuation, thus making it impossible to create terms that are only punctuation. There are many test results that are only punctuation. This annotator was created specifically, for this task to pick up such entities.

2.13 Person Tokens Annotator

This annotator identifies persons in the text.
Note: The BTRIS data has persons already redacted, so this annotator is not useful currently and was turned off for this work.

2.14 Slot: Value Repairs

There are various failings of the current slot:value annotator that these corrective annotators fix, using downstream annotations not available to the slot:value annotator when it runs in the sequence in the pipeline. This annotator fixes some of the failures that are fixable.

2.15 CCDA Section Header Annotator

This annotator creates section headers based, for the most part, on dictionary lookup. The annotator uses an augmented version of HL7's list of approved section headings. The list was augmented a lot for this task because OT/PT specific sections do not appear within the CCDA domain (yet).

2.16 CCDA Panel Section Header Annotator

Panels are sections within clinical documents that list test results for blood tests, primarily. This annotator creates headers for panel sections. *Note: Panels are ignored for this work, and this annotator is turned off.*

2.17 CCDA Section Annotator

This annotator creates section zones from the end of the section name down to just before the beginning of the next section name.

2.18 Sentence Section Repair

This is a corrective annotator. Once section headings are determined, there is need to adjust (erroneous) sentence boundaries to exclude section names.

2.19 Quoted Utterance Annotator

This annotator creates quoted text. Symptoms are typically found in “quoted text”, so it’s useful to find them.
Note: Quoted text does not play a role in the Body Function task and though it is on, this feature is not used downstream.

2.20 Sentence Repairs

This is a corrective annotator. This annotator removes lists that only have one element to them and turns those back into sentences. Sentences that end with a number also caused issues because the numbers look like list delimiters. Lists that have list delimiters like “1. 2.” that have the list delimiter ordering out of order are likely not lists, but sentences that end with numbers. Sentences that have tabs in them are likely to be from multi-column formats, where, within the process of OCRing them, the OCR software injected tabs to indicate a new column.

2.21 Assertion Annotator

This annotator, part two of the two assertion annotators, creates assertion attributes to all annotations based on the assertion evidence noted from the assertion evidence annotator.

2.22 Section Name in Terms Attribute Annotator

It is useful to know what section a term is mentioned in. This is useful to filter out mentions found that come from sections you do not care about. This annotator adds the section name to each term in the document. This is done outside the term annotator, which happens before the section zones are computed.

3. Body Function Pipeline Components

The body function pipeline’s purpose is to identify Body Function mentions. That is, an utterance that includes a body location, a body function type (such as strength, range of motion or reflex) along with some kind of qualifier related to the body function type. The body function pipeline has been appended to the pipeline that decomposes the text into sections, sentences, slot:values, lists, phrases, terms, and tokens.

The body function pipeline relies upon having terms in the document already looked up and classified prior to the next set of annotators and knowing what sections those terms occurred in.

3.1 Body Location Annotator

The Body Function Location annotator transforms terms that have Body Location as one of the categories (body location, body laterality) from the Body Location Lexicon into a Body Location label. This annotator will filter out mentions that are not about the patient/subject, or is a mention that is attributed to the patient (subjective statement), or is historical. This annotator filters out mentions within sections that were identified as needing to be ignored.

3.2 Body Strength Annotator

The Body Strength annotator transforms terms that have Body Strength as one of the categories from the Body Strength Lexicon into a Body Strength label. This annotator will filter out mentions that are not about the patient/subject, or is a mention that is attributed to the patient (subjective statement), or is historical. This annotator filters out mentions within sections that were identified as needing to be ignored.

3.3 Body Range of Motion Annotator

The Body Range of Motion annotator transforms terms that have Body Range of Motion as one of the categories from the Body Range of Motion Lexicon into a Body Range of Motion label. This annotator will filter out mentions that are not about the patient/subject, or is a mention that is attributed to the patient (subjective statement), or is historical. This annotator filters out mentions within sections that were identified as needing to be ignored.

3.4 Body Reflex Annotator

The Body Reflex annotator transforms terms that have Body Reflex as one of the categories from the Body Reflex Lexicon into a Body Reflex label. This annotator will filter out mentions that are not about the patient/subject, or is a mention that is attributed to the patient (subjective statement), or is historical. This annotator filters out mentions within sections that were identified as needing to be ignored.

3.5 Body Function Qualifier Annotator

Body Function Qualifier Annotator looks for qualifiers within the sentence/utterance scope of utterances that have body function evidence in it (strength, ROM, reflex). The qualifier identification looks for terms with qualifier categories (fair, normal etc.). or patterns that include numbers and units of measure that are indicative of the body function type. Since similar qualifiers are found near confounding entities like pain, balance and coordination, such qualifiers, when found around terms that have categories of pain, balance and/or coordination get filtered out. Multiple qualifiers within the same scoping window get merged into one mention.

3.6 Body Function (Mention) Annotator

This annotator looks for evidence of a body function including Strength, ROM, Reflex, location and quantification within a sentence/utterance scoping window. A Body Function Mention entity is made from the maximal span of that evidence.

3.7 Body Function Qualifier Polarity Annotator

When there is a qualifier within a body function mention, this annotator will create an attribute to characterize the qualifier as -1 (below normal function), 0 (ambiguous), or 1 (at or above normal function). Each body function type has rules that determine how those values are assigned.
